# Laboratory and field studies to investigate the efficacy of a novel, orally administered combination product containing moxidectin, sarolaner and pyrantel for the prevention of heartworm disease (*Dirofilaria immitis*) in dogs

**DOI:** 10.1186/s13071-019-3702-6

**Published:** 2019-09-11

**Authors:** Kristina Kryda, Robert H. Six, Kelly F. Walsh, Susan J. Holzmer, Sara Chapin, Sean P. Mahabir, Melanie Myers, Tammy Inskeep, Jady Rugg, Blair Cundiff, Aleah Pullins, Michael Ulrich, John W. McCall, Tom L. McTier, Steven J. Maeder

**Affiliations:** 1 Veterinary Medicine Research and Development, Zoetis, Inc., 333 Portage St, Kalamazoo, MI 49007 USA; 2 Cheri-Hill Kennel and Supply Inc., 17190 Polk Road, Stanwood, MI 49346 USA; 3 TRS Labs Inc., 215 Paradise Blvd, Athens, GA 30607 USA

**Keywords:** *Dirofilaria immitis*, Heartworm, Prevention, Macrocyclic Lactone, Sarolaner, Moxidectin, Pyrantel, Laboratory study, Field study, Dog

## Abstract

**Background:**

*Dirofilaria immitis* is a filarial parasite of dogs that can cause serious or fatal cardiopulmonary disease. Three studies were conducted to evaluate the efficacy and safety of monthly treatment with moxidectin in a chewable tablet product in combination with sarolaner and pyrantel to prevent heartworm disease in dogs after experimental challenge and in a clinical field study in the USA.

**Methods:**

In two laboratory studies, dogs (8 per group) that had been inoculated 30 days prior with 50 third-stage *D. immitis* larvae were randomized to treatment on Day 0 with placebo or combination product, at the minimum dose of 24 µg/kg moxidectin, 2 mg/kg sarolaner and 5 mg/kg pyrantel (as pamoate salt). Study 2 also included groups treated with tablets containing moxidectin-alone (24 µg/kg) or sarolaner-alone (2 mg/kg). Efficacy was evaluated ~ 5 months after inoculation by adult heartworm counts at necropsy. In the field study, 410 dogs ≥ 8 weeks-old from 23 USA veterinary clinics were treated for 11 months with either combination product at 24–48 µg/kg moxidectin, 2–4 mg/kg sarolaner and 5–10 mg/kg pyrantel (*n* = 272) or Heartgard® Plus (ivermectin/pyrantel) at the label recommended dose rate (*n* = 138). Efficacy was evaluated on Day 330 using antigen and microfilaria testing to assess adult heartworm infection.

**Results:**

In the laboratory studies, there were no heartworms recovered from any dog treated with the combination product or moxidectin alone and all dogs treated with placebo or sarolaner-alone were infected with 20–44 adult heartworms. In the field study, all dogs treated with the combination product tested negative for heartworm infection on Day 330, whereas two dogs treated with Heartgard® Plus tested positive. The Heartgard® Plus-treated dogs that tested heartworm positive were from the lower Mississippi River Valley region, where heartworm resistance has been confirmed to occur. The combination product was well tolerated in all studies.

**Conclusions:**

In laboratory studies, no heartworms were recovered from dogs treated with a single dose of the novel combination product containing moxidectin, sarolaner and pyrantel. Additionally, in the field study no dog tested positive for adult heartworm infection when dosed with the combination product monthly for 11 months, while two dogs treated with Heartgard® Plus tested positive.

## Background

Heartworm (*Dirofilaria immitis*) is a filarial parasite transmitted by mosquitoes that can cause severe and life-threatening disease in dogs and other animals [1, 2]. The disease can be prevented by prophylactic treatment with macrocyclic lactones (MLs) and year-round administration is recommended [3–5]. These compounds are administered monthly in oral (e.g. ivermectin, milbemycin oxime) or topical (e.g. moxidectin, selamectin) formulations, or as an injectable extended-release formulation (moxidectin) that provides 6- or 12-months protection from heartworm disease [5]. Reports of lack of efficacy of MLs as a heartworm preventative have increased and the causes are multifactorial and include poor owner compliance, incorrect dose rate and frequency, timing and interpretation of heartworm diagnostic tests and the heartworm test sensitivity and specificity [6–9]. More recently, heartworm strains with lowered susceptibility or resistance to ML preventives have been identified [10–13]. Research indicates that prophylactic efficacy may also depend on the active ingredient in the formulation and the dosage regimen selected [13–15].

Moxidectin is a ML used for heartworm prevention with a variety of dose rates, formulations and routes of administration. An oral tablet formulation of moxidectin dosed at 3 µg/kg, administered once, was 100% effective against susceptible strains but incompletely effective (19–62% efficacy) against some ML-resistant strains [13]. However, an extended-release, injectable formulation providing 170 µg moxidectin/kg (ProHeart® 6, Zoetis) was highly effective (99.5%) against the JYD-34 ML-resistant strain when inoculated with third-stage larvae two days after treatment [16]. ProHeart® 6 or a similar formulation dosed at 500 µg/kg moxidectin (ProHeart® SR-12, Zoetis) provided complete protection after a single injection against susceptible strains when inoculated with third-stage larvae at 365 days after dosing [17–19]. Moxidectin applied topically at 2.5 mg/kg (in combination with imidacloprid, Advantage Multi®, Bayer) was shown to be completely effective in one study against infection with ML resistant JYD-34 heartworms 30 days after a single treatment [20].

Based on its proven efficacy against *D. immitis*, moxidectin was selected for inclusion in the oral chewable tablet combination formulation in addition to sarolaner and pyrantel. Importantly, moxidectin is included in the combination product at a minimum dose of 24 µg/kg (range, 24–48 µg/kg). Moxidectin alone administered orally to dogs at this dose rate provided improved protection against heartworm strains determined previously to be resistant to MLs [21].

Besides heartworm, dogs are also affected by several parasites that can cause serious, even fatal disease and which can transmit a number of pathogens and zoonotic agents. The blood-feeding activity of external parasites such as fleas and ticks cause direct irritation and in heavy infestations may lead to anemia and even death [22, 23]. Additionally, fleas and ticks can transmit a number of disease organisms that can cause severe, even life-threatening illness in both dogs and humans [24, 25]. Sarolaner belongs to a potent new class of ectoparasiticides (isoxazolines) and when administered as a single-entity (Simparica®, Zoetis) has been shown to provide fast and consistent efficacy against ectoparasites in dogs and cats [26–28]. Roundworms and hookworms commonly infect the gastrointestinal tract of dogs and are potentially zoonotic [29]. Pyrantel alone and in combination with other parasiticides has been shown to be effective for the treatment and control of adult roundworms and hookworms [30].

Current veterinary practice guidelines in the USA advise year-round prevention, control and/or treatment of most if not all of these common parasites of dogs [3, 31]. A broad-spectrum parasiticide treatment that could provide this desired level of protection would offer a convenient all-in-one option for dog owners. Consequently, a novel oral chewable tablet combination formulation that includes moxidectin, sarolaner and pyrantel is being assessed for prevention of heartworm and lungworm disease, and to control flea and tick infestations for one month and treat and contol roundworms and hookworms.

Here we report on the studies conducted to evaluate the efficacy and safety of this novel combination product administered as an oral chewable tablet for the prevention of heartworm disease in dogs in laboratory and field clinical settings.

## Methods

### Laboratory studies

Two masked, negative placebo-controlled, randomized, laboratory efficacy studies were conducted. Study procedures were carried out in accordance with the CVM Guidance for Industry #111, Effectiveness of Anthelmintics-Specific Recommendations for Canine (GL19) [32] and complied with Good Clinical Practice guidelines [33]. The first study (Study 1) was conducted at a test facility in Michigan, USA, to confirm efficacy of the combination product. The second study (Study 2) was a similar dose confirmation study conducted in Georgia, USA, but included additional groups treated with some of the individual formulation components to characterize their contribution, if any, to the efficacy of the combination product. To ensure masking of the study, all personnel conducting observations, animal care or performing infections and parasite recovery and counts were masked to treatment allocation.

#### Animals

Male and female purpose-bred Beagle dogs that had been reared in a mosquito-proof facility were used for these studies. In Study 1, 16 dogs approximately 7 months of age and weighing 8.2–10.8 kg were included in the study. Thirty-two dogs approximately 5 months of age and weighing 6.1–9.8 kg were enrolled in Study 2. Dogs were identified by uniquely numbered ear tattoos and housed individually in indoor enclosures in mosquito-proof facilities that conformed to accepted animal welfare guidelines and ensured no direct contact between dogs. Dogs were fed an appropriate ration of a commercial, dry, laboratory, canine feed for the duration of the study. Water was available *ad libitum*. All dogs were given a physical examination to ensure that they were in good health prior to inclusion in the study. General health observations of each dog were performed at least once daily throughout the study.

#### Design

Day 0 was designated as the day that dogs received the experimental treatment. All dogs were inoculated with *D. immitis* on Day -30. The studies followed a randomized complete block design. The dogs were ranked by Day -2 body weights into blocks, and within each block dogs were randomly allocated to treatment with either placebo or the combination product (Study 1), or in Study 2, to treatment with placebo, the combination product, moxidectin-alone or sarolaner-alone resulting in eight dogs in each treatment group. Necropsy for *D. immitis* recovery was conducted on Day 122 (152 days post-inoculation) in Study 1 and on Day 118 (148 days post-inoculation) in Study 2.

#### Strains and infection

The *D. immitis* strains used in these studies had been obtained from infected dogs in the field (within 3 years prior to study initiation). Study 1 used the ZoeKY-2013 strain collected from a naturally occurring case of canine heartworm in Kentucky in 2013. The third-stage larvae were inoculated into laboratory dogs and validated as an infective strain through diagnosis of circulating microfilariae and positive heartworm antigen tests. The GCFL-01-2014 strain used in Study 2 was originally collected from a naturally occurring field case of canine heartworm from Florida in 2014. Dogs were inoculated with third-stage larvae and the strain was validated as a successful infective strain through positive antigen test and by detection of circulating microfilariae. Both of these strains have been characterized as not resistant to MLs [13].

On Day -30, each dog was inoculated with 50 viable third-stage larvae of the respective *D. immitis* strain by subcutaneous injection in the inguinal region.

#### Treatment

On Day 0, dogs were dosed with either placebo tablets containing inert formulation ingredients (vehicle), combination product chewable tablets (Study 1 and 2) or tablets containing sarolaner-alone or moxidectin-alone (Study 2). Tablets, of varying strengths were used such that a combination of tablets could be administered to ensure dogs were appropriately dosed to the minimum end of the proposed dose range. Each dog received from one to three tablets containing the combination product to provide as close as possible the minimum target dose of 24 µg/kg moxidectin (actual doses ranged from 24 to 29 µg/kg), 2 mg/kg sarolaner (actual doses ranged from 2.0 to 2.5 mg/kg), and 5 mg/kg pyrantel (as pamoate salt) (actual doses ranged from 5.1 to 6.3 mg/kg) or the equivalent number of placebo tablets based on pre-treatment body weights. In Study 2 the additional groups were similarly dosed with tablets containing only sarolaner (minimum target dose of 2 mg/kg; actual doses ranged from 2.0 to 2.5 mg/kg) or moxidectin (minimum target dose of 24 µg/kg; actual doses ranged from 26 to 30 µg/kg). Placebo and active tablet presentations were similar in appearance to maintain masking. Feed was withheld from dogs at least 12 h prior to treatment to 4 h post-treatment. All doses were administered by hand-pilling to ensure accurate dosing. Each dog was observed for several minutes after dosing to ensure that the dose was swallowed and for up to 2 h post-dosing for any signs of emesis. Dogs were examined for general health at least once daily and any reactions to treatment at 1, 3, 6 and 24 h after treatment.

#### Heartworm tests and evaluation

Blood samples from each animal were collected in potassium EDTA tubes on Days -32 and 90. These samples were examined for adult *D. immitis* antigen [Solo Step®, Heska, CO (Study 1); DiroCHEK®, Zoetis, NJ (Study 2)] and for microfilariae (modified Knott’s test, both studies) to confirm no pre-existing heartworm infection. The Day 90 blood examination was conducted to detect heartworm infections that may have been acquired prior to selection for the study that were not detectable by the testing performed on Day -32.

All dogs were humanely euthanized on Day 122 in Study 1 and Day 118 in Study 2. At the time of euthanasia, each dog was given 1–2 ml of heparin (1000 USP units/ml) intravenously prior to a lethal dose of an approved euthanasia agent. After euthanasia, the pleural and peritoneal cavities were examined for adult *D. immitis* worms, and the posterior and anterior venae cavae were clamped before removal of the heart and lungs. The precava, right atrium, right ventricle and pulmonary arteries (including those coursing through the lungs) were dissected and examined for worms. The number and viability of worms recovered from each dog was recorded.

#### Statistical analysis

The individual dog was the experimental unit. Prior to statistical analysis, total viable worm counts were natural log-transformed [log_e_(x+1)]. The mixed linear model contained the fixed effects of treatment and the random effects of room, block within room, and error. Geometric mean worm counts (back-transformed means) were calculated from the least squares means (SAS Version 9.3 or higher, Cary NC). Treatment differences were assessed at the two-tailed 5% level of significance (*P* < 0.05). Percent reduction *vs* placebo in total worm count for each treatment group was estimated using the following formula:

$$\% {\text{ reduction}} = 100 \times \left[ {{\text{mean count }}\left( {\text{placebo}} \right){-}{\text{mean count }}\left( {\text{treated}} \right)} \right]/{\text{mean count }}\left( {\text{placebo}} \right)$$

### Field study

**Fig. 1:**
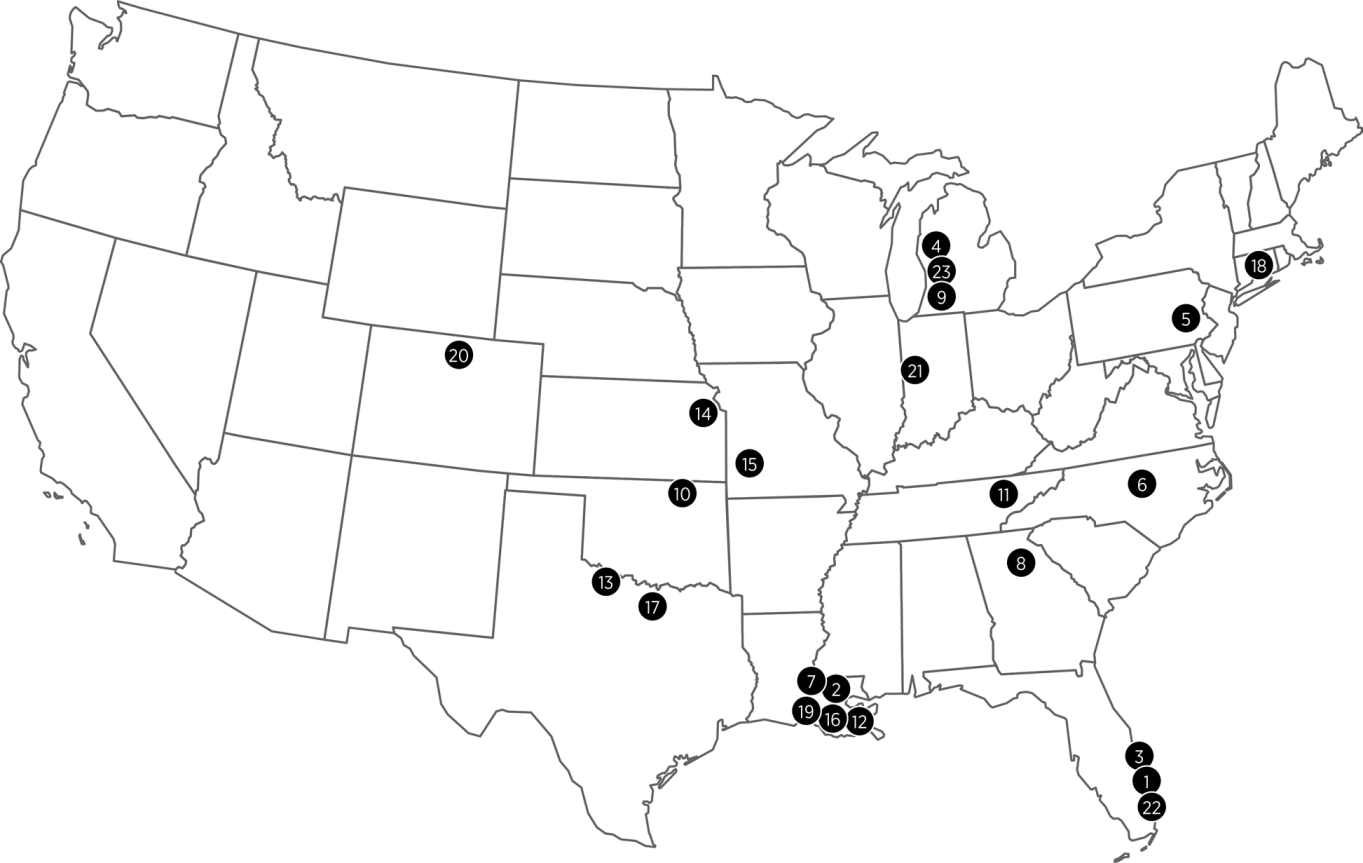
Location of 23 veterinary clinics in a field study investigating the efficacy of a combination product containing moxidectin, sarolaner and pyrantel

The clinical field study was conducted using client-owned dogs enrolled from 23 veterinary clinics in diverse, heartworm endemic regions of the USA. Sixteen of the 23 veterinary clinics were in the mid-south/southeastern USA, with 5 of the 23 clinics in the lower Mississippi River Valley (LMRV) region, an area that includes large portions of Louisiana, Mississippi and Arkansas, western portions of Tennessee and Kentucky and southern portions of Missouri and Illinois (Fig. 1). The study design was a randomized, single-masked, multi-center clinical study using Heartgard® Plus (Boehringer-Ingelheim) as a positive control. The study complied with Good Clinical Practice guidelines [33].

#### Animals

Patients were selected from dogs presenting to veterinary practices from a diverse range of households and living conditions representing the range of client-owned dogs typical in heartworm endemic regions of North America. Only one dog from each household could be enrolled and there was no limit on the numbers of pets in a household. Dogs had to be at least 8 weeks of age and weigh at least 1.3 kg. There were no breed or sex restrictions, but dogs intended for breeding or that were pregnant or lactating were not eligible for enrollment. Dogs had to be amenable to handling for the study observations and had owners capable of administering the oral medications. Dogs with stabilized, pre-existing conditions under veterinary management and who were expected to be able to survive the duration of the study could be included, but dogs with existing uncontrolled medical conditions that might confound the study were excluded. All dogs 6 months or older were confirmed to be negative for adult heartworm antigen and circulating blood microfilariae. Animals that had been treated with injectable moxidectin (ProHeart® 6, Zoetis) in the prior 12 months or that were older than 6 months of age and that tested positive for heartworm infection or circulating microfilariae (including *Dirofilaria* spp. and *Acanthocheilonema* spp.) could not be enrolled. Dogs could not be included if they had been recently treated with a heartworm preventative and were within the labeled “protective period” for that product at the start of the study. Dogs suspected to have previously had adverse reactions to the positive control product were also excluded.

#### Design

The study utilized a randomized complete block design with dogs randomly allocated in order of presentation to the clinic in a 2:1 ratio to treatment with the combination product or the positive control product. Dogs were kept under their normal home conditions for the duration of the study.

At the initial screening visit, dogs were weighed, given a physical examination, had blood collected for hematology, blood chemistry and heartworm (antigen and microfilaria) testing, and had urine collected for urinalysis. All personnel conducting observations or animal care, or performing parasitological assessments were masked to treatment allocation. Designated unmasked personnel (Dispenser) at each clinic were solely responsible for the allocation and dispensing of the experimental products and instruction of the owners. The Dispenser discussed flea and tick control with the owners of dogs in the positive control group and, if it was needed, NexGard® (afoxolaner, Boehringer-Ingelheim) was provided.

Combination product chewable tablets were supplied in six different strengths to provide the targeted dose range of 24–48 µg/kg moxidectin, 2–4 mg/kg sarolaner, and 5–10 mg/kg pyrantel (as pamoate salt). Commercial Heartgard® Plus (ivermectin plus pyrantel, Boehringer-Ingelheim) chewables were dispensed per label directions. Owners were provided with the treatments and instructed on dosing methods and observations while at the veterinary clinic, and then they administered the tablets and evaluated product consumption at home once monthly for 11 months. Day 0 was defined as the day on which the dog received its first dose. The dose could be offered at any time of the day, with or without food. Unmasked study personnel from the clinic contacted the owner within 2 days of dispensing to ensure the treatment had been administered successfully and determine if any adverse events had been noted.

All dogs were subsequently presented to the clinics on Days 30, 60, 90, 120, 150, 180, 210, 240 and 330 with a target window of ± 3 days. At each visit, dogs were weighed and examined for general health. Owners were dispensed the appropriate tablets to be dosed in the home environment as described above. On Day 240, owners were provided three doses to allow for continued monthly dosing (i.e. Days 240, 270 and 300) until the dog came in for the final scheduled clinic visit on Day 330. On Days 120 and 240, all dogs had blood collected for heartworm antigen and microfilaria testing. On Day 330 (study completion), dogs were weighed, given a complete physical examination, had blood collected for hematology, blood chemistry and heartworm (antigen and microfilaria) testing, and had urine collected for urinalysis. Any dogs presented for an unscheduled visit during the study were examined by a veterinarian for any abnormal health issues. Any dog withdrawn from the study prior to completion was given a physical examination and had samples collected for clinical pathology. Clinical pathology, heartworm antigen tests (DiroCHEK®, Zoetis, NJ) and modified Knott’s tests for microfilariae were conducted by Marshfield^TM^ Labs, Marshfield, WI.

#### Statistical analysis

The individual dog was the experimental unit. Heartworm antigen and microfilaria test results were summarized in two-way frequency tables to show the number of animals in each combination (for *D. immitis* antigen and blood microfilaria) of the test results. The effectiveness of the combination product was evaluated based on *D. immitis* antigen and blood microfilaria test results on Day 330. Non-inferiority of the combination product to the control product for the prevention of heartworm infection was tested at the one-sided 0.025 significance level using an equivalence margin of 5% using exact confidence limits for comparing the difference in prevention rates based on whether dogs were positive for at least one heartworm test on Day 330 using StatXact 10 software.

## Results

### Laboratory studies

#### Study 1

All animals were dosed uneventfully; no tablets were expelled during or after dosing and no emesis of tablets was noted during post-dosing observations. No adverse events were observed during the study; however, one combination product-treated dog was noted with a single instance of diarrhea on Day 0 prior to treatment, that resolved uneventfully.

On Days -32 and 90, all dogs tested negative for *D. immitis* antigen and microfilariae, confirming that none of the dogs were infected with *D. immitis* prior to inoculation for the study.

On Day 122, adult *D. immitis* were recovered from all eight placebo-treated dogs. Counts ranged from 20 to 37 worms per dog, and geometric (arithmetic) mean counts were 30.7 (31.1) (Table 1). No adult *D. immitis* were recovered from any combination product-treated dog (*P* < 0.0001).

**Table 1:** Total worm counts, means and percent reduction relative to placebo for dogs treated orally with combination product tablets 30 days after experimental infection with *Dirofilaria immitis* (ZoeKY-2013, Laboratory Study 1)

	Treatment	
	Placebo	Moxidectin/Sarolaner/Pyrantel combination product
Total worm counts for individual dogs	32	0
	32	0
	32	0
	36	0
	20	0
	37	0
	33	0
	27	0
Range	20–37	0–0
Arithmetic mean	31.1	0
% reduction	–	100
Geometric mean	30.7	0
% reduction	–	100
*P*-value	–	< 0.0001

#### Study 2

All animals were dosed uneventfully; no tablets were expelled during or after dosing and no emesis of tablets was noted during post-dosing observations. The only adverse health observations were those minor ailments typically observed in laboratory dogs, such as diarrhea, conjunctivitis/cherry eye, minor injuries, dermatitis and alopecia. These occurred before and after treatment and the incidence was similar for both treatment groups. There were no adverse reactions to treatment with the combination product or the single component sarolaner and moxidectin tablets.

On Days -32 and 90, all dogs tested negative for *D. immitis* antigen and microfilariae confirming that none of the dogs were infected with *D. immitis* prior to inoculation for the study.

On Day 118, adult *D. immitis* were recovered from all placebo-treated dogs. Counts ranged from 35 to 44 worms per dog, and geometric (arithmetic) mean counts were 39.9 (40.0). As expected, adult *D. immitis* were recovered from all eight sarolaner-treated dogs. Counts ranged from 32 to 40 worms per dog, and geometric (arithmetic) mean counts were 36.7 (36.8) (Table 2). Percentage reduction in geometric (arithmetic) mean count in the sarolaner-treated dogs compared to placebo was 8.2% (8.1%) and mean count was lower than placebo (*P* = 0.0026). No adult *D. immitis* were recovered from dogs treated with the combination product or moxidectin-alone. The geometric mean total adult *D. immitis* counts for the combination product and moxidectin-alone treated groups were significantly lower than placebo and sarolaner-alone (*P* < 0.0001). There was no difference among the counts for the dogs treated with the combination product and moxidectin-alone (*P* > 0.05).

**Table 2:** Total worm counts, means and percent reduction relative to placebo for dogs treated orally with combination product, sarolaner-alone or moxidectin-alone tablets 30 days after experimental infection with *Dirofilaria immitis* (GCFL-01-2014, Laboratory Study 2)

	Treatment			
	Placebo	Sarolaner	Moxidectin	Moxidectin/Sarolaner/Pyrantel combination product
Total worm counts for individual dogs	39	37	0	0
	42	33	0	0
	44	32	0	0
	41	40	0	0
	39	39	0	0
	40	38	0	0
	40	38	0	0
	35	37	0	0
Range	35–44	32–40	0–0	0–0
Arithmetic mean	40.0	36.8	0	0
% Reduction	–	8.1	100	100
Geometric mean	39.9	36.7	0	0
% Reduction	–	8.2	100	100
*P*-value *vs* placebo	–	0.0026	< 0.0001	< 0.0001
*P*-value *vs* sarolaner	–	–	< 0.0001	< 0.0001
*P*-value *vs* moxidectin	–	–	–	1.0

### Field study

#### Demographics

The patient populations of the two treatment groups were similar (Table 3). Of the total dogs, 50.2% were males and 49.8% were females. About 80% of animals in both groups were neutered. At the time of enrollment, dogs ranged in age from 8 weeks to 13 years and the mean age of the dogs was similar for both groups. Purebred dogs comprised about 60% of the enrolled population with Labrador Retrievers, Golden Retrievers, German Shepherds, Yorkshire Terriers, Chihuahuas, Pit Bulls, Shih Tzus, Boxers and Australian Shepherds being enrolled most frequently. Living conditions for the dogs were similar for both groups; about 62% spent time primarily indoors and about 33% spent time both indoors and outdoors, and the remainder (~ 5%) were primarily outdoors.

**Table 3:** Details of client-owned dogs enrolled in a clinical field study investigating the efficacy and safety of combination product chewable tablets administered orally once a month for 11 months for the prevention of heartworm disease in the USA

Category	Treatment group		
	Moxidectin/ Sarolaner/Pyrantel combination product	Heartgard® Plus	Total
No. of females (intact/spayed)	139 (24/115)	65 (11/54)	204 (49.8%)
No. of males (intact/neutered)	133 (36/97)	73 (15/58)	206 (50.2%)
Mean initial age in years (range)	4.12 (0.15–13.0)	4.70 (0.25–13.0)	4.32 (0.15–13.0)
Pure-bred/mixed breed (%)	58.1/41.9	58.7/41.3	58.3/41.7
No. indoors and outdoors (%)	91 (33.5)	44 (31.9)	135 (32.9)
No. mostly indoors (%)	169 (62.1)	86 (62.3)	255 (62.2)
No. mostly outdoors (%)	12 (4.4)	8 (5.8)	20 (4.9)

#### Evaluable cases

A total of 410 client-owned dogs from 23 clinics were enrolled from 1 June 2015 to 2 July 2015 and were included in safety evaluations (Table 4) with 272 dogs receiving the combination product and 138 dogs allocated to the Heartgard® Plus (ivermectin + pyrantel) positive control. A total of 365 dogs (246 combination product, 119 Heartgard® Plus) completed the study and were included in the evaluation of efficacy. Twenty-one dogs (15 in the combination product group and six Heartgard® Plus-treated) were withdrawn from the study prior to Day 330. The most common reason for withdrawal was owner discretion (e.g. owner no longer able or willing to participate in the study; 8 dogs). Six dogs were withdrawn due to owner non-compliance, 3 dogs due to adverse events not related to treatment with test product and 4 dogs due to ‘other’ reasons, such as unrelated or pre-existing medical issues or owner death. The other 24 dogs (11 in the combination product group and 13 in the Heartgard® Plus-treated group) were excluded from the efficacy evaluation because of protocol deviations that could have impacted the interpretation of data (most commonly incorrect or missed dosing).

**Table 4:** Clinic location and number and percentage of dogs enrolled in a clinical field study investigating the efficacy and safety of combination chewable tablets administered orally once a month for 11 months for the prevention of heartworm disease in client-owned dogs presented in the USA

Clinic location	Moxidectin/Sarolaner/Pyrantel combination product		Heartgard® Plus		Total	
	*n*	%	*n*	%	*n*	%
Lake Worth, FL	12	4.4	6	4.3	18	4.4
West Palm Beach, FL	8	2.9	4	2.9	12	2.9
Boca Raton, FL	8	2.9	4	2.9	12	2.9
Bogart, GA	14	5.1	7	5.1	21	5.1
Baton Rouge 1, LA	9	3.3	4	2.9	13	3.2
Baton Rouge 2, LA	8	2.9	4	2.9	12	2.9
Livonia, LA	13	4.8	6	4.3	19	4.6
Metairie, LA	10	3.7	5	3.6	15	3.7
Zachary, LA	10	3.7	5	3.6	15	3.7
Wichita Falls, TX	8	2.9	4	2.9	12	2.9
Grapevine, TX	10	3.7	5	3.6	15	3.7
Lawrence, KS	8	2.9	4	2.9	12	2.9
Bartlesville, OK	20	7.4	10	7.2	30	7.3
Fort Collins, CO	10	3.7	5	3.6	15	3.7
Terre Haute, IN	11	4.0	6	4.3	17	4.1
Farragut, TN	10	3.7	6	4.3	16	3.9
Raleigh, NC	12	4.4	6	4.3	18	4.4
Springfield, MO	26	9.6	14	10.1	40	9.8
Quakertown, PA	23	8.5	12	8.7	35	8.5
Chester, CT	10	3.7	5	3.6	15	3.7
Caledonia, MI	12	4.4	6	4.3	18	4.4
Grand Rapids 1, MI	10	3.7	5	3.6	15	3.7
Grand Rapids 2, MI	10	3.7	5	3.6	15	3.7
Total	272	100	138	100	410	100

#### Heartworm evaluations

All dogs in both treatment groups were negative for *D. immitis* antigen and blood microfilariae in blood samples collected on Days 0, 120 and 240, confirming that none were infected prior to initiation of study treatment. On Day 330, all of the combination product-treated dogs were negative for *D. immitis* antigen and blood microfilariae. Two of the Heartgard® Plus-treated dogs were positive for *D. immitis* antigen, and one of these dogs also had circulating microfilariae (Table 5).

**Table 5:** Test results from a clinical field study investigating the efficacy and safety of combination product chewable tablets administered orally once a month for 11 months for the prevention of heartworm disease in the USA

Treatment group	Study day	Adult HW antigen test				Microfilaria test			
		Negative		Positive		Negative		Positive	
		No. of dogs	%	No. of dogs	%	No. of dogs	%	No. of dogs	%
Moxidectin/Sarolaner/Pyrantel combination product	330	246	100	0	0	246	100	0	0
Heartgard® Plus	330	117	98.3	2	1.7	118	99.2	1	0.8

The development of *D. immitis* infection in these two Heartgard® Plus-treated dogs could not be explained by errors in dosing. Dosing records for both dogs confirmed that the correct dose of Heartgard® Plus was administered to each dog at the correct time throughout the study and that no scheduled doses were missed. Both dogs were located in Livonia, LA in the LMRV.

Statistical analysis of the field study data for whether an animal was positive for at least one heartworm test on Day 330 demonstrated that the combination product was non-inferior to Heartgard® Plus at an equivalence margin of 5% at the one-sided 0.025 level of significance (*P* < 0.0001), and it also had a significantly different prevention rate at the 2-sided 0.05 level of significance (*P* = 0.0424).

#### Health observations

Over the 11-month study period, all abnormal health events, irrespective of their causality, duration, or severity, were reported in 69.1% of dogs treated with the combination product and in 60.1% of dogs treated with Heartgard® Plus. The abnormal clinical signs observed were typical of those expected to occur in the general dog population and occurred with similar frequency in both treatment groups. Adverse events occurring in 2.0% or more of treated dogs in one or both treatment groups included vomiting, diarrhea, lethargy, anorexia, polyuria, hyperactivity and polydipsia.

Serious adverse events occurred in dogs in both treatment groups (7 dogs that received combination product; 5 dogs that received Heartgard® Plus). In the combination product group, these events included three dogs with seizure activity, one dog with primary pulmonary neoplasia, one dog with worsening hind-limb paresis, one dog with necrotizing meningoencephalitis along with renal disease and one dog with a splenic mass that was euthanized due to worsening chronic renal failure. In the Heartgard® Plus group there were two dogs with seizure activity and one dog that was euthanized due to the diagnosis of neoplasia. Based on clinical examinations, history and timing of events, these serious adverse events for both treatment groups were considered unrelated to the experimental treatments. Additionally, in the Heartgard® Plus group there were two dogs that were heartworm-positive.

The mean body weights and weight changes were similar for both groups. In both groups, from Day 0 to Day 330, the mean body weight in dogs ≤ 6 months of age increased as would be expected for a group of growing animals (combination product, 12.1 to 26.2 kg; Heartgard® Plus, 10.2 to 21.5 kg). For dogs 6 to 12 months of age, the mean body weight increased slightly (19.1 to 21.6 kg; 20.1 to 22.7 kg, respectively) and for dogs over 12 months of age the mean body weight remained relatively constant (21.7 to 21.9 kg; 20.2 to 19.5 kg, respectively).

Mean results for post-treatment hematology and serum chemistry in both treatment groups were similar and within the normal reference range. The results of urinalyses were similarly unremarkable in both treatment groups.

Various concomitant medications and therapies were administered to dogs during this study and these were consistent with the demographic and study duration for the veterinary patient population examined. The most common administered concomitant medications included those typically used in general veterinary practice: immunologicals; antibacterials; anti-inflammatories; psycholeptics used for sedation and anesthesia; other anesthetics and analgesics; ectoparasiticides, insecticides and repellents which were mostly used in the Heartgard® Plus-treated group as it did not provide ectoparasite control. All concurrently used medications and therapies were well tolerated.

## Discussion

Resistance of heartworms to ML preventatives has been confirmed in both phenotypic efficacy studies and by genetic characterization of individual strains [10, 11, 13, 20, 34, 35]. Most of these strains are from the LMRV of the USA [35]. Every currently marketed compound (i.e. ivermectin, milbemycin oxime, moxidectin and selamectin) has been shown to be less than 100% efficacious against a resistant strain(s) in at least one controlled study [10, 14, 20, 36]. This includes Advantage Multi®, Bayer (imidacloprid + moxidectin) applied topically 30 days post-inoculation with the JYD-34 ML-resistant strain, in which one of the eight treated dogs had two adult heartworms at necropsy (McCall, unpublished data, 2013). New treatment options are needed to effectively protect dogs from this threat. In laboratory efficacy studies, moxidectin has demonstrated the highest potency of all the MLs tested as an oral preventative against *D. immitis*. Moxidectin administered at a dose of 0.5 µg/kg 60 days following inoculation of L3 *D. immitis* was 100% efficacious [37]. In comparison, in separate studies, ivermectin was 100% efficacious at a dose of 2 µg/kg when given 30 days post-inoculation, while milbemycin oxime provided 100% efficacy at 0.5 mg/kg dosed at 60 days post-inoculation [38–40]. The high *in vivo* potency of moxidectin is likely due to its unique physiochemical and pharmacokinetic properties [41, 42]. Moxidectin is more lipophilic and generally has a longer elimination half-life and larger volume of distribution than ivermectin [43, 44]. These properties result in extensive tissue distribution, particularly to fat, and a long residence time [41]. With the sustained levels of drug in tissue and plasma, moxidectin is available to target migrating stages of parasites such as heartworm [42]. In laboratory efficacy studies to investigate the optimal dose of moxidectin, it was shown that increasing both the dose and the number of consecutive monthly doses of moxidectin results in increased protection against ML-resistant *D. immitis*. Most notable in these studies, three consecutive doses at 24 µg/kg provided ≥ 98.8% efficacy (4 out of 5 dogs protected) against three heartworm strains determined previously to be resistant to MLs (JYD-34, ZoeMo and ZoeLA) [21]. Additional consecutive doses may provide improved efficacy as previously demonstrated for other MLs with multiple consecutive monthly dosing regimens [45]. For this reason, moxidectin at the minimum dose of 24 µg/kg (range 24–48 µg/kg) was included in the combination product and subsequently evaluated in clinical field and laboratory efficacy studies.

In two laboratory studies and a clinical field study the efficacy and safety of a combination product for the prevention of heartworm infection in dogs was evaluated. In the two laboratory studies, experimental heartworm infections were successfully induced in all 16 placebo-treated dogs, while none of the sixteen dogs that were dosed with the combination product at 24 µg/kg moxidectin, 2 mg/kg sarolaner and 5 mg/kg pyrantel (as pamoate salt) at 30 days post-inoculation developed heartworm infections. Additionally, in the second study, the eight dogs treated with sarolaner-alone at 2 mg/kg all developed heartworm infections similar to placebo-treated dogs, whereas none of the dogs treated with moxidectin-alone at 24 µg/kg developed heartworm infections. This confirms that moxidectin is the component of the combination product that provides heartworm prevention. There were no adverse reactions to treatment with either the combination product or single component tablets.

In the field study none of the 246 client-owned dogs that were dosed monthly with the combination product at the dose range of 24–48 µg/kg moxidectin, 2–4 mg/kg sarolaner and 5–10 mg/kg pyrantel (as pamoate salt) for 11 months tested positive for adult heartworm infection (adult *D. immitis* antigen or blood microfilariae) at the end of the study. It is notable that the two dogs in the Heartgard® Plus (ivermectin/pyrantel) positive control group tested positive for heartworm infection on Day 330. Both were positive for adult *D. immitis* antigen, and one of these dogs also had circulating microfilariae. It is possible that the absence of microfilariae seen in the modified Knott’s tests of one case was due to the timing of infection or the dog being infected with female worms only [46]. These positive cases confirm that these two dogs were exposed to heartworm infected mosquitoes during the study and validates that the study design (one year duration of the study, blocking and random allocation of treatment at each clinic) provided the potential for exposure of the patient population to infected mosquitoes, thus confirming the validity of the field study. In the field study, a number of dogs were enrolled in the Southeast USA (Fig. 1) with approximately 68% (167 combination product and 82 Heartgard® Plus dogs) from the mid-south/southeastern USA of which 64 dogs (44 combination product and 20 Heartgard® Plus) were from the LMRV. Both heartworm-positive dogs were from the LMRV which is where the majority of reports of resistant heartworm are clustered [6, 13]. As the dosing records confirmed, both dogs were dosed correctly, and because one of these dogs had circulating microfilariae despite monthly treatment, it is possible that these two dogs were infected by a ML-resistant strain of *D. immitis*. Another recent clinical field study evaluating the efficacy of ProHeart® 12 (*n* = 236), also using Heartgard® Plus (*n* = 218) as the positive control, yielded similar results with none of the dogs treated with ProHeart® 12 testing positive for adult heartworm infection (adult *D. immitis* antigen or blood microfilariae) at the end of the study, while four Heartgard® Plus-treated dogs also from the LMRV tested positive [19].

Safety was also evaluated in this study with more than 2800 doses of the combination product administered to more than 270 dogs. The adverse events noted during the study were typical of the conditions expected in any general dog population and were similar to those seen with the commercial positive control product (Heartgard® Plus). The health observations reported in these studies were not unexpected as the single components of the combination product have been commonly used and/or are well characterized in dogs. Pyrantel as a single-entity has been safely used for nematode control for many years in a variety of species [47]. Sarolaner as a single-entity, oral ectoparasiticide has demonstrated safety for dogs in clinical field studies and under conditions of normal field use [48, 49]. The oral moxidectin dose used in the combination product is higher than that used previously for oral heartworm prevention [50]. Moxidectin alone or in combination with imidacloprid has been studied in the most sensitive population, and oral doses up to 250 µg moxidectin/kg were well tolerated [51–53]; this dose corresponds to 5-fold the highest end of the dose range of moxidectin in the combination product that was used in the clinical field study, 48 µg/kg.

## Conclusions

In two laboratory studies, using recently collected field strains, a single oral dose of combination product chewable tablets providing moxidectin at the minimum recommended dose of 24 µg/kg at 30 days after inoculation with third-stage larvae of *D. immitis* prevented the development of heartworm infection. Additionally, in the one of the laboratory studies, treatments with moxidectin or sarolaner alone confirmed that heartworm prevention was solely due to the moxidectin component. In the field study conducted in heartworm endemic areas of the USA, none of the combination product-treated dogs tested positive for adult heartworm infection when dosed monthly for 11 months. By contrast, in the positive control group using the commercial product (Heartgard® Plus, ivermectin/pyrantel) two dogs tested positive for adult heartworm infection. The combination product was well tolerated in all three studies.

## Data Availability

The dataset supporting the conclusions of this article is included within the article.

## Abbreviations

CVM: Center for Veterinary Medicine, US Food and Drug Administration
HW: heartworm
L_3_: third-stage larvae
LMRV: lower Mississippi River valley
ML: macrocyclic lactone
